# *‘I don’t know when it’s my turn’*: reasons for low uptake of cancer-related healthcare services in Germany

**DOI:** 10.1186/s12889-025-23656-6

**Published:** 2025-07-19

**Authors:** Ali Zafar, Milena Koch, Luis Weller, Nelli Tschobur, Andreas Ihrig, Till Johannes Bugaj, Gwendolyn Mayer, Franziska Baessler

**Affiliations:** 1 Center for Psychosocial Medicine, Heidelberg University Hospital, Thibautstrasse 4, 69115 Heidelberg, Germany; 2 Heidelberg Academy of Sciences and Humanities, Heidelberg, Germany; 3 Medical School Hamburg, University of Applied Sciences and Medical University, Hamburg, Germany

**Keywords:** Cancer, Screenings, Health seeking behavior, Public health, Behavioral science, Barriers

## Abstract

**Background:**

Participation rates in early cancer detection and follow-up cancer related healthcare services are lower than anticipated in many high-income countries. Germany also provides a variety of these services free-of-charge under its highly subsidized public healthcare system. This qualitative study aimed to determine the barriers perceived or experienced by service users in Germany against utilizing cancer screenings and treatment services.

**Methods:**

A semi-structured interview protocol was developed based on current literature and in consultation with stakeholders in cancer-related professions. The interviews aimed at exploring the underlying reasons for underutilization of cancer prevention or treatment services. Interviews were conducted from a pool of respondents (*N* = 57) from an earlier phase of the study based on voluntary response sampling. All interviews were transcribed verbatim from audio recordings and transcripts were processed in MAXQDA 2022. Two authors analyzed the qualitative data using hybrid inductive-deductive coding methodology described by Udo Kuckartz.

**Results:**

Overall 23 interviews were conducted. The participants included 13 current or former cancer patients and 10 non-patients [male = 14, female = 9; mean age = 58.65 years (SD = 13.79)]. Lack of awareness and absence of reminders emerged as the most common reason for not attending cancer screenings or treatment services. Most participants described lack of information regarding the availability or timing of the recommended screenings as their main reasons. Other predominant barriers against accessing cancer-related healthcare services were associated with time constraints and work responsibilities, unavailability of physicians and appointments, administrative bureaucracy, fear of negative news, pain or side-effects, low personal relevance, norms of gendered behavior, feelings of shame and insecurity, and communication difficulties with practitioners.

**Conclusions:**

Most pertinent barriers were rooted in lack of information and awareness about existing cancer-related healthcare services among participants. Our findings suggest that tailored interventions that address systemic, personal and social factors are essential for improving screening uptake. A proactive approach by healthcare personnel and health insurance companies for providing concise and timely information on cancer-related offers to service users is recommended to improve participation rates.

**Supplementary Information:**

The online version contains supplementary material available at 10.1186/s12889-025-23656-6.

## Introduction

Cancer is the second leading cause of death in Europe with about 20% of annual deaths attributed to various types of cancer [1]. The cancer burden continues to grow, implying enormous physical, emotional and financial strain on individuals, families, communities and health systems. However, survival rates are improving because of better accessibility to healthcare services, early detection of cancer and advancements in treatment and care [2]. Early diagnosis and screenings have consistently demonstrated improved clinical outcomes, especially for breast, colorectal and recently lung cancers [3, 4]. 

Cancer-related care in Germany is part of a broad national prevention strategy for non-communicable diseases and is covered under social health insurance [5]. Screenings for cervical, breast, skin, prostate and colorectal cancer are available free-of-charge for eligible populations mainly determined by age and sex [6]. The first screening for women is recommended for cervical cancer at the age of 20 years and continuing every year until the age of 34 years. From 35 years onwards, pap smears are offered every three years. The screening for breast cancer by palpation is recommended yearly from 30 years onwards while mammography scans are offered every two years for women aged between 50 and 75 years. Skin cancer screenings are recommended for men and women from the age of 35 years onwards every two years. For men aged 45 years, prostate cancer screening are suggested every year while screenings for colorectal cancer (FOBT/colonoscopy) start from 50 years onwards [6, 7]. Formal invitations are only sent out to individuals eligible for mammography and colorectal cancer screenings while service users mainly rely on healthcare providers to inform and recommend screenings for cervix, skin and prostate during their routine visits [8].

Despite their benefits, early detection and screening services remain underutilized in Germany as well as other high-income countries. The standardized nationwide skin cancer screening program introduced in Germany in 2008 was utilized by less than half of the eligible participants [9–12]. In other surveys, about two-thirds of the women reported using recommended offers for breast cancer screening while colonoscopy offers were utilized by about 50% of the eligible participants [13, 14]. Similarly, in the US, about 13% of people eligible for lung cancer screening utilized the service [15], 69% utilized the recommended colorectal cancer screening [16], 73% of those eligible took the cervical cancer screening [17], while 76% utilized breast cancer screening [18]. In the Netherlands, the number of participants in the population screening programs for breast cancer, cervical cancer and colorectal cancer has declined for several years, according to the Dutch National Institute for Public Health and the Environment [19].

 Socioeconomic disadvantages such as migration background, lower incomes, lower education, and infrastructural deficiencies such as distance from facilities, poor transport services, lack of insurance as well as lack of healthcare facilities are known as major barriers against cancer-related care [20–24]. Further studies have reported on the strong relationship between public healthcare expenditure and improved cancer outcomes in high-income countries [25, 26], which aim to overcome disparities in healthcare access by providing free or subsidized healthcare, routine screening offers, and accessible healthcare facilities. Several theories and frameworks have been proposed to predict and understand individual health-seeking behaviors. For instance, the Health Belief Model (HBM) is widely used in public health to predict individual intentions for health-related behaviors [27]. The normative contentment theory suggests that society and culture in general normalize poor health behaviors and outcomes, especially among men [28]. Since cancer screenings are prospective measures before the manifestation of symptoms or disease, it is difficult to explain their uptake with health-seeking frameworks alone. Moreover, these theories and models not only put the onus of responsibility on the service users, but they also do not account for infrastructural or socioeconomic factors inhibiting or promoting health-seeking behavior. Sociologists have criticized such models for ‘victim blaming’ the service users and characterizing individuals as passive victims of disease [29].

To understand the low attendance rates for available cancer-related health services in Germany, this qualitative study aimed to identify the barriers perceived or experienced by service users. We employed a qualitative approach to explore the underlying personal beliefs and perceptions that influence the decision-making process of individuals in cancer-related healthcare. Qualitative interviews are useful to explore the views, experiences, beliefs and/or motivations of individuals on specific matters (e.g. factors that influence attendance at the health practitioner). They provide a deeper understanding of the social phenomena than would be obtained from purely quantitative methods such as questionnaires [30]. Since earlier studies in Germany have either focused on a single type of cancer or on a specific section of the population [9–14], we employed a wide-ranging, all-inclusive approach to investigate all factors of participation for recommended screenings (due to age/gender) or specified treatments (due to symptoms).

## Methods

This qualitative study is based on analysis of semi-structured interviews with participants selected via voluntary response sampling [31]. The interviews aimed to determine the barriers against attending cancer prevention or treatment services faced by the respondents in Germany. The participants provided their informed consent for voluntary interviews and agreed to anonymous publication of results in the first phase of the research project, which was conducted between September 2021 and September 2022. The study was approved by the ethics committee of the University of Heidelberg Medical Faculty (S-538/2021). The study was conducted in accordance with the Declaration of Helsinki [32] and is reported in line with the COnsolidated criteria for REporting Qualitative research (COREQ) checklist (supplementary material) [33].

### Interview protocol

Semi-structured interviews were conducted in German language by one female author (MK), who was trained in ethnographic research methods and had several years of experience in social work. One-on-one interviews were conducted with participants either via telephone or online video calls, primarily because of the COVID-19 pandemic-related safety and hygiene measures. The interviews were held between January 2022 and February 2023. The interviews lasted between 30 and 60 min and were recorded on audio devices with the consent of the participants. There were no repeat interviews with the participants and transcripts were not returned to participants for proofreading.

The interview protocol was informed by the Health Belief Model [27] and Walter’s Model of Pathways to Treatment [34]. The primary questions were finalized after two Delphi group discussions [35] among cancer-related service providing stakeholders, including medical doctors, psychologists, psycho-oncologists, and nursing and social healthcare professionals. After two rounds of discussions, comments and adaptions, the questions were finalized with 100% consensus between the stakeholders and the research team.

The interview protocol consisted of three sections. In the first section, participants were asked to talk openly about their perceived or experienced barriers to cancer screenings and/or treatment services. This question served as “icebreaker” meant to build rapport with the interviewee. It also served as a guide for the interviewer about which barriers might be important for the individual and might require an in-depth consideration during the course of the interview.

In the second section, the participants were shown a list of the major barriers reported in literature and the first study phase, wherein a cross-sectional quantitative instrument was used to determine the main barriers reported by the German population. They were asked to deliberate upon the three most relevant factors for themselves. In the third section, the participants were asked to explain the reasons for choosing their three barriers and the possible contributing factors (specific experiences or perceptions) for selecting these particular barriers.

Only the primary questions were the same for all participants and the structure and script were modified and adapted throughout the fieldwork, especially after the first few interviews. Moreover, for participants with a past or present cancer diagnosis, the interviewer followed up with different secondary questions than for non-patients for identifying barriers against screenings and treatment offers.

### Participants

In the quantitative stage of the research project, 57 out of 314 participants, including current or former cancer patients and non-patients, had provided contact details for taking part in a voluntary interview. Emails were sent out to these participants, inviting them to an online interview. The research questions and objectives were explained to the participants in the invitation email. Overall 20 individuals did not respond at all to the invitation emails while 11 participants did not want to be interviewed anymore. Two email addresses were unreachable, whereas one participant did not appear on the day of the interview. After two reminder emails in two weeks to unresponsive participants, the interviews were called off due to time constraints and personnel management.

### Data analysis

For qualitative analysis, the interview recordings were transcribed verbatim by three authors (MK, LW & NT) before processing. All interview transcripts were proofread and redacted for clarity before processing in the software. Data were analyzed with MAXQDA (Version 2022).

The transcripts were coded using the hybrid coding methodology (deductive-inductive) described by Kuckartz and Raediker [36], which is suitable for systematically analyzing complex qualitative data. This method enabled us to not only address our predefined research questions but also allowed for the discovery of new themes. The Kuckartz’s method by its structured and iterative nature allows data to be organized into hierarchical categories, thereby supporting transparency and rigor. Primary (first-level) codes were created deductively informed by the questions of the semi-structured interview protocol. The interviews transcripts were then inductively coded (secondary codes) line by line independently by two authors (LW & NT). Whenever necessary, up to third-level subcategories were introduced for better clarity and structure. Intercoder reliability was established through consensus-building and interviews were re-read and re-coded if necessary. After final discussion between authors, the inductive secondary codes were assigned to the primary coding categories developed deductively through an iterative, triangulating process.

For the sake of clarity and readability, we only report the codes with highest prevalence and those most pertinent to the research question in this publication. Moreover, only the example quotes used in this publication were translated from German into English language. The complete coding protocol is available in the supplementary material (in German).

## Results

Overall 23 interviews (male = 14; female = 9) were conducted. The youngest participant (male) was 23 years old and the oldest was 82 years old (mean age = 58 years). The participants included 13 current or former cancer patients (male = 7; female = 6) and 10 non-patients (male = 7; female = 3). For better readability, the responses were processed into patient and non-patient groups. After descriptive analysis and deductive coding, we were able to broadly assign most barriers into three macro-level categories of: (a) systemic barriers, which were related to inefficiencies within the healthcare system; (b) personal barriers, which were factors related mostly to individual beliefs, behaviors or attitudes; and (c) sociocultural barriers, which related loosely to social norms or to communication with health professionals. An additional theme of COVID-19-related barriers emerged since our study took place during the course of the pandemic, which had severely disrupted healthcare services in Germany between 2020 and 2022.

### Systemic inefficiencies

#### Absence of precise information

The most common complaint among participants was about absence of information regarding the availability or timing of the recommended cancer screenings. They lacked awareness about the screenings available for them or the time period personally relevant for them to attend the services. The interviewees claimed they were never asked by doctors or health authorities to attend screenings nor were they sent any reminders by their health insurance companies. One patient claimed he never knew when he needed to do a colon cancer screening while another said he was never told to attend any screenings:

*“…I didn’t know and still don’t know when it’s my turn for a colon cancer screening.” (patient*,* male*,* 55).*

*“…I have two friends*,* one has quite advanced prostate cancer with surgery*,* the other has bladder cancer and they only went when they were in pain… but we’d never been asked to go for a screening.” (non-patient*,* male*,* 64)*.

A common misperception was that screenings were relevant only for the older population and an intervention was required only when there were some symptoms. Similarly, ambiguous information and uncertainties regarding the costs of cancer screenings and treatments were described as frustrating. The participants were unsure if the costs for such services were covered or not by the insurance companies. The fear of bearing these immense costs was spoken of as a constant burden in the decision-making process, as described by a female participant:

*“Yes*,* the financial aspect*,* the fact that many people were initially afraid of the costs involved in the major interventions. You don’t know*,* up to that point you hadn’t dealt with it at all. You usually hear about it from friends*,* neighbors or something like that and there is often also a lack of knowledge […].” (patient*,* female*,* 62).*

The responsibility of finding out relevant information regarding cancer-related health services appeared to be a personal initiative for the interviewees, e.g. by researching about cancer screening on the internet, suggesting that practitioners or health insurance companies did not provide them information:

*“[…] I also researched […] on the internet and also looked at what was common before. As I said*,* I knew about this program in Rheinland-Pfalz. There is actually a kind of aftercare pass*,* which I also got for myself […].” (patient*,* female*,* 62).*

#### Time constraints and work priorities

Not having time for attending their recommended interventions emerged as another commonly perceived factor for not attending cancer-related services. Many participants described problems with finding enough time to think about attending screenings during routine life and work. Attending a screening was described as a time-consuming process, firstly for finding an appropriate appointment with a clinic of choice as well as taking time off work, which was perceived as a higher priority by working employees, as suggested by a male participant:

*“As an employee*,* you often don’t have the time. Because you simply have to get an appointment with the few doctors that are available - it’s even worse now in times of COVID-19. You have to get the time in your job or find time off work*,* which is often not even possible.” (patient*,* male*,* 53).*

For one patient, the effort involved in planning or attending cancer screening and/or treatment also played an important role in the decision-making process, not only about organizing the journey but also the time spent in the waiting room:

*“So then I have to worry: now I have to make another appointment*,* then I need a chauffeur*,* then I drive in again for ages*,* then I sit in a huge crowd of people*,* I then wait maybe 2–3 hours until it’s my turn and then the practitioner looks at it and says: ‘Oh no*,* that’s not so bad*,* you don’t need to do anything’. And goodbye. And that could be so much easier for cancer patients to sort out and without them having such a burden*,* stress*,* pressure […].” (patient*,* female*,* 52).*

For many interviewees, thinking about health, and cancer screenings in particular, assumed a lower priority because of the stress about first finishing daily activities related to work and life. For example, for two male participants attending screenings just did not fit into a regular schedule:

*“[…] sometimes you just have your daily stress […] and you say to yourself: “Oh no*,* I’ll do it next week”*,* because it wouldn’t affect me for the time being […] if I didn’t go to the doctor.” (non-patient*,* male*,* 35).*

“*[…] and time in any case. So I think that many people are simply so involved in all sorts of things that they don’t even get to think about the fact that they might need to have a screening examination. (patient*,* male*,* 63).*

#### Shortage of physicians and appointments

Complaints about lack of appointments for cancer-related services were widespread. The participants recalled having difficulties consistently in getting appointments with doctors, particularly specialists, because of few healthcare personnel, especially in rural regions. A male patient complained that he received an appointment with a dermatologist nine months in advance:

*“In the countryside*,* you have one dermatologist for the whole district. […] Get an appointment with a dermatologist here - lasting three quarters of a year! If you get an appointment at all […].” (patient*,* male*,* 53).*

Another participant believed that new patients with public health insurance did not receive appointments easily with local doctors, who gave them appointments with considerably longer waiting times, as compared to their routine patients.

*"[…] that’s just the thing when you want to go to a specialist as a statutory health insurance patient. First you have to find someone and then you look for someone on the internet or ask friends or family members. Then you’re immediately told that if you’re not a patient there*,* they won’t have any appointments that year anyway or that we’re not taking on any new patients anyway. I think that’s an enormous*,* enormous hurdle.” (non-patient*,* female*,* 62).*

#### Administrative bureaucracy

Administrative processes, particularly related to compensation by statutory health insurance, were described as leading to delays or postponement of cancer screenings or treatments. Many participants complained about the time-consuming process of completing paperwork or getting actual information out of health insurance companies if an intervention was covered or not. These bureaucratic hurdles with hospitals and insurance companies resulted in burdening the already struggling patients. One participant found the paperwork extremely cumbersome:

*“[…] all these forms and all these applications for admission to hospital*,* then to the health insurance companies. I often hear that it would also be or is a problem. So you have to work your way through it and I think […] that’s a real burden.” (patient*,* female*,* 62).*

Several interviewees described the process of reimbursement of costs by health insurance companies as challenging. For cancer screening, one participant mentioned the so-called IGeL services (Individuelle Gesundheitsleistungen or Individual Health Services that are not included under statutory health insurance in Germany) as a financial barrier:

“*Money was sometimes one of those things*,* so many things are now IGeL services for statutory health insurance patients*,* that’s certainly a problem.” (patient*,* male*,* 63).*

*“[…] so being ill is also really expensive*,* […] I’m lucky that I can pay for it*,* […] but still this costs this and that and now I have to discuss it with the insurance company*,* or I have to apply for this and that and then argue with them three more times’.” (patient*,* female*,* 33).*

#### COVID-19 pandemic-related barriers

Many participants described postponing their appointments for cancer screening or follow-up cancer care due to the extraordinary restrictions in place during the COVID-19 pandemic. The pandemic also negatively affected the care of cancer patients due to contact restrictions, fear of getting an infection as well as lack of appointments.

*“During COVID-19*,* I actually didn’t go twice because I was there once and I thought*,* I’m guaranteed to get infected*,* it’s so crowded*,* I was really scared*,* especially because I now had these cancer recurrence […].” (patient*,* male*,* 63).*

*“[…] the hospital actually assigned someone to provide social care […] the lady only did that (the consultation) very briefly on the phone and […] referred me to an association.” (patient*,* male*,* 55).*

### Personal behaviors

#### Fear of negative news, pain or side-effects

The fear of negative news regarding a possible cancer diagnosis was among the most frequently perceived reasons among personal behaviors for not attending screenings. Even with family history of cancer and awareness about screening offers, one participant talked about her hesitancy to attend recommended screenings and avoiding them until she noticed some symptoms.

*“[…] probably an important point*,* is worrying about negative news…we’ve had a lot of experience with cancer in our family and therefore*,* I think it’s always like*,* yes*,* oh*,* you have to go there now and who knows what will turn up and as long as I don’t have any problems*,* it’s no big deal.” (patient*,* female*,* 33).*

Many interviewees expressed concerns about the safety of cancer screenings or side-effects of treatment procedures. They expressed uncertainties about the procedures and concerns about negative physical side effects (especially in the context of cancer treatment (e.g. chemotherapy)). However, it was not necessarily perceived as a reason for not attending cancer-related health services, but rather an accompanying ‘necessary evil’. Apart from the fear of undergoing the procedure as well as the physical side effects, a previous negative experience, for instance the fear of pain and emotional distress made some interviewees uncomfortable about attending future services. A female participant complained about the painful mammography keeping her away from breast cancer screenings:

*“[…]the mammography examination with this device*,* I found it so extremely painful and*,* I would say*,* somewhat degrading that I did not have breast cancer screening for a while because of it.” (non-patient*,* female*,* 62).*

A patient who had undergone colon surgery in the past also complained about being scared of painful potential consequences of a colonoscopy:

*“[…] my body formed a stoma through the scar*,* and […] after a year […] everything was fine again*,* […]*,* but I was just so scared of this screening examination (colonoscopy)*,* […] I was afraid that something would get hurt.” (patient*,* female*,* 73).*

#### Low personal relevance

Conversely, many interviewees believed that cancer screenings were not relevant for them because there were no symptoms present or that they were too young to go to screenings since cancer was perceived as a disease for the elderly.

*“I feel healthy*,* so I can postpone it*,* nothing will happen.” (patient*,* female*,* 52).*

Even at an older age, a lack of symptoms led to a disregard for screenings, citing low personal relevance or a lack of understanding on the importance of early detection procedures, as understood from this description by a male participant:

*“[…] I put it off (the cancer screening) […] a bit. Well*,* on the one hand it was […] a time problem […] and I think awareness was still a bit lacking. […] I think you have to be a certain age to see that it makes sense.” (non-patient*,* male*,* 57).*

On the other hand, the presence of cancer patients or survivors in the immediate social environment of the participants and encouragement from friends or family seemed to facilitate the uptake of screenings through raising personal awareness.

*“[…] a neighbor who has now had colon cancer. Then you see for yourself the need to perhaps do it after all […].” (non-patient*,* male*,* 57).*

### Sociocultural internalization

#### Norms of gendered behavior

A gender-specific understanding of societal roles especially among men emerged as a reason for lower uptake of cancer screening services. The male gender and its social role as appeared resistant towards the topic of cancer because illness and visits to the practitioner made men appear “weaker” in the eyes of their friends or colleagues. A male participant pointed out the aspects of traditional masculinity associated with men’s health and the association of medical problems as weaknesses:

“*[…]men are brought up to be strong*,* not to whine*,* to perform and not to show weakness […]at my age or in my social network*,* you have erectile dysfunction or prostate cancer or… then of course that’s a… brutal weakness […].” (non-patient*,* male*,* 64).*

Another indirect effect of the societal gender role norms was lower perceived levels of personal risk among male participants. Similarly, the perception of indispensability at work and a lack of feeling responsible for their own health were also mentioned by male participants:

I could imagine that men believe more that nothing can happen to them. That’s such a macho attitude. None of this can happen to me. Women are probably a bit more sensitive (090140RB, patient, male, 82).

*“I think they tend to have the feeling ‘I’m irreplaceable here’ […] it’s like ‘I can’t be replaced here*,* I absolutely have to’.” (patient*,* male*,* 65)*.

*“[…] at least in my generation*,* and men are simply not brought up to take on responsibility for others or for themselves. […].” (non-patient*,* male*,* 64).*

#### Shame and insecurity

Feelings of shame and insecurity made some participants feel uncomfortable during medical examinations, particularly when the health practitioner was from the opposite gender and the examinations involved physical touching such as skin cancer screening. A female participant claimed feeling comfortable with female physicians and recalled that her husband felt extremely embarrassed during his skin cancer screening with a female doctor:

*“Well*,* I generally only see female doctors*,* as a woman. My husband also made a skin cancer screening appointment and ended up with a woman. Of course*,* he went through with it anyway*,* but he was so embarrassed afterwards. Next time he would have made sure that he went to a man.” (patient*,* female*,* 42).*

Such feelings were more specific to individuals visiting the services for the first time or initial stages. Conversely, after visiting these services several times over the years made it easier to navigate the services and interactions with the physicians.

“*And shame of course*,* for me of course no longer after six years*,* when you know how the whole thing works*,* but when you go to such examinations for the first time*,* of course there is always shame and uncertainty*,* nobody can tell me that this is not the case. You only get over it when you know exactly how it all works and when you’ve been to lots of screenings. (patient*,* male*,* 62).*

Similar social norms also included the taboo surrounding the topic of cancer in society, which leads to people not getting involved with the topic of cancer and cancer screening services:

*“[…] there’s often a taboo […] I mean*,* death and illnesses like that are taboo anyway. And come along with something like that and let’s say you have cancer and are trying to get a job*,* everyone is terribly sorry […].” (patient*,* male*,* 70).*

#### Communication difficulties with practitioners

For many participants, communication problems with health practitioners were important when making decisions about visiting cancer-related services. The interviewees complained about a hierarchy during interactions with physicians whereby they could not speak to them as equals and perceived that they were being patronized, especially when they had difficulties in understanding the medical terminologies used by the practitioner.

“*[…]it didn’t actually have anything to do with being asked whether you really wanted this treatment. And in the beginning I actually found this hierarchy thing quite difficult at times.” (patient*,* female*,* 33).*

“*[…] I actually thought that I could use the language fluently*,* but this terminology is also unknown to me*,* I had to read it up again and again or look up what it actually means*,* what was said there […].” (patient*,* male*,* 63).*

Other communication-related barriers mentioned by the participants were the practitioner’s lack of empathy and the lack of time to talk with the patient, especially when the physicians did not take enough time to explain how cancer screenings and/or cancer treatment worked.

In one case, taking initiative in holding the dialogue and asking questions from the practitioners until all clarifications were provided was suggested as a work around for avoiding communication problems.

*“I don’t let communication problems arise because I ask questions until I fully understand. And I also tell someone who speaks to me in their medical language that I don’t understand them and I ask as long as I can understand them as a normal patient.” (non-patient*,* female*,* 62).*

## Discussion

In this qualitative study, we explored the underlying reasons behind low participation rates for early detection and other cancer-related healthcare services in Germany. Using semi-structured interviews, we assessed the barriers perceived and experienced by service users that influenced their decisions and behaviors about attending cancer-related health offers. The most pertinent barriers described by the interviewees were assigned into three macro-level categories of (a) systemic inefficiencies within the German healthcare system; (b) personal beliefs, behaviors or attitudes; and (c) internalized social norms and communication problems. These findings suggest that despite their usefulness psychological frameworks like the HBM are unable to fully capture the effects of structural and sociocultural variables on personal behaviors. The HBM was originally proposed in the 1950 s when participation rates in tuberculosis screening remained low despite their increased accessibility and convenience [37]. The case of cancer screenings is similar in high-income countries that continue to report declining cancer screening utilization, suggesting that social determinants of health (SDOH) remain relevant to improve quality of healthcare delivery and outcomes [38]. For instance, high household income has been associated with higher participation rates in using early detection cancer tests across Europe [39]. Despite Germany’s high-income status and socialized healthcare system, service providers are encouraged to look for social risk factors and address the identified needs by informing service users about availability and accessibility of resources.

### Systemic inefficiencies

In our study, we interpreted most of the perceived and experienced barriers against cancer-related healthcare services in Germany as systemic inefficiencies of the healthcare system. The most widespread complaints were regarding a general lack of information or ambiguous information about the available services or their coverage under health insurance. Many participants simply did not know about the existing offers and complained about a lack of reminders from general practitioners or health insurance companies. Lack of information has been reported as a major factor for lower attendance rates of cancer screenings [40–42]. In a study in Germany, almost half of the respondents stated not knowing about available skin cancer screening [12]. Public awareness campaigns by general practitioners or medical organizations could be promoted as feasible interventions for providing information about screenings to health service users.

Poor knowledge about cancer and screenings is also a known factor for underutilization of cancer-related offers among men [43, 44]. For men in Germany, this may derive from the later initiation of screenings as their first cancer screening (skin) becomes available at the age of 35 years. Whereas, for women cervical cancer screenings are available from 20 years onwards [7]. A 2013 report by the Robert Koch Institute (RKI) had reported higher awareness among women (88.5%) than men (75.7%) [45]. Participation rates increased significantly among older men, suggesting a correlation between older age and higher risk awareness about services and attendance [45]. As interactions of patients with physicians generally increase with age, more exposure with healthcare providers might act as a facilitator for participation in later years.

For improving participation rates, it is advisable that healthcare professionals not only introduce service users to cancer-related information at an earlier age but also to remind them about available services. Physician-patient discussions about screening tests are noted among the strongest predictors of cancer screening uptake [46, 47]. The existing system appears to put the responsibility of finding relevant information on the service users, who are already paying for these services in advance under the German taxation system. Instead of personal initiatives by service users, the onus of providing correct and concise information regarding cancer-related services to individuals rests with the healthcare professionals to avoid any misinformation, uncertainties and reservations. German health insurance companies, which maintain databases of their customers, should be proactively reminding service users about available services and inviting them at the recommended ages.

A lack of time in routine working life was another reason perceived by many participants for not attending cancer-related health services. This is consistent with an earlier study in Germany, in which over one-fourth of female participants complained about time problems for their inability to attend mammography appointments even after receiving written invitations [48]. In a randomized controlled trial, appointments with fixed date and time for non-attenders of breast screening significantly improved participation [49]. Doctor appointments are not considered working time according to German laws and employees get paid time off only in cases where medical care is necessary. Moreover, regular healthcare facilities, apart from hospital emergencies, are closed outside of routine working hours on weekdays as well as on entire weekends. Since cancer screenings do not fall under necessary care, service users have no choice but to take off time during work for a medical check-up. Moreover, the entire process of getting an appointment for a routine check-up and then requesting absence at work can be cumbersome for most working people, considering the physical and psychological preparation involved in planning or attending cancer screenings.

These time-related barriers when coupled with lack of personnel and appointments, administrative red tape and lengthy waiting times, require attention for initiating patient-centered interventions. Potential solutions to these issues could be centralized health facilities where screening services are provided under one roof and modifications in the legal regulations allowing employees to attend screenings without taking a day off or unpaid leave. However, a lack of time could also be interpreted as a personal barrier stemming out of health-related priorities and their influences on psychological wellbeing.

Several COVID-19-related barriers were also reported as the pandemic had disrupted cancer-related services for over two years when the German healthcare system came under extraordinary pressure. Although the country fared better than other European countries, the pandemic severely impacted the care of cancer patients. Many participants complained about postponing their appointments for cancer screenings or follow-up care due to social distancing restrictions, fear of getting infected as well as lack of appointments. Similar observations were made in other countries because of the unprecedented circumstances during the COVID-19 pandemic [50–52].

### Personal behaviors

Several important reasons pertaining to reluctance or avoidance of cancer-related healthcare services could be attributed to personal behaviors, individual beliefs and attitudes. Many service users described feelings related to fear of receiving news of developing cancer or concerns related to safety of cancer screenings and side-effects of treatment procedures. These findings are supported by previous studies that report fear of a positive cancer diagnosis or its consequences as major barriers against seeking medical advice or interventions [53–55]. In one survey, men with a lower perception of fear were twice more likely to attend a screening as compared to men who were more fearful [56]. Earlier studies also suggest that even when significantly distressed, many cancer patients do not seek psychological help [57–59], mostly confirming the role of personal attitudes as central barriers [60, 61]. Psycho-oncologists have observed typical behaviors of repression and defense mechanisms among cancer patients for either ignoring their disease by not thinking about the symptoms, which in turn helps them to continue their routine life, or by refusing to acknowledge having the disease [62–64].

Fear of cancer or screenings may be justified in many cases because a positive cancer diagnosis is indeed a life-altering experience. However, this fear might also be a subconscious manifestation of a lack of awareness about cancer, leading to a limited understanding of its causes, symptoms or morphology of cancer. Many participants in our study perceived the screenings to be irrelevant without symptoms or for younger people that could be attributed to their lack of awareness in the absence of information from physicians and insurance companies. This is evident from a few examples, who were motivated by the presence of cancer patients or survivors in their social circles to attend cancer screenings. These findings reiterate the importance of open communication between physicians and service users with the intent of informing and educating them about the risks and benefits of cancer screenings and treatment procedures.

### Sociocultural internalization

Despite higher incidence and mortality rates from cancer than women [1, 65], men are known to visit physicians or attend cancer screenings less often than women [44]. Since our sample was skewed towards male participants, a major reason for underutilization of cancer-related services emerged to be the social norms of gendered behavior. The role of men in society as strong masculine providers appeared to influence the decision-making process because being sick meant men appeared “weaker” in their social circles. Earlier studies have reported on traditional masculinity as a barrier for men such as their reluctance to speak about cancer, not asking for help, embarrassment in talking about symptoms, stubborn behavior and fear of stigmatization at work or family [53, 55, 66, 67]. Our findings highlight the need for innovative communication strategies targeted at raising cancer awareness especially among men. It is known that men who have in-depth discussions with their physicians report significantly less difficulties in navigating the healthcare system [68], reiterating the need for open discussions between physicians and service users.

Feelings of shame, insecurity and hierarchy in communication with physicians also emerged as powerful factors for our participants’ reluctance to attend cancer-related services. Some of the participants felt uncomfortable with physicians of the opposite gender while others complained about perceptions of lack of empathy and lack of time to talk with the patient. Communication problems with health practitioners are a common theme and patient-centered approaches in health services research. Aspects of communication are important factors during decision-making about visiting cancer-related services and many studies have reported on patients concerns about clearly expressing their problems during a consultation [69, 70]. Another study among cancer patients and survivors reported on issues with providers’ behavior and the consultation environments as influential barriers for service users during their visits [71]. These factors are important cues of patient-centered care for physicians, who have been accused of not giving enough time to patients [72]. Our findings suggest that persistent inquiries by physicians and informative discussions with service users may be necessary for improving participation rates for screenings.

### Limitations

Some limitations in the study design and our results are noteworthy. Firstly, the voluntary response sampling method leaves considerable room for selection bias and may not reflect the opinions of the general population. Unfortunately, because of the low response rate, the sample became highly skewed towards male participants and current or former cancer patients, especially for whom experiencing the disease is a clear motive for adopting preventive attitudes and behaviors. However, we interviewed all the participants who responded to our invitation emails. Due to time and personnel constraints, we did not pursue the participants who did not respond to invitation and reminder emails.

The interviews for his study were conducted between January 2022 and February 2023, when Germany was still recovering from the COVID-19 pandemic. As the participants were talking about events in the recent past, they might have exaggerated some aspects of facing barriers in accessing cancer-related healthcare services. During the interviews, the interviewer observed that the participants instead of reporting their own subjective feelings often shared the experiences and perceptions about other people within their social environments. This became evident as a majority of our participants claimed to have regularly utilized the recommended cancer prevention and/or treatment services. Therefore, results regarding the barriers against these services may have limited generalizability on the general public behavior.

The knowledge bias based on age and gender was unavoidable in this study because men in Germany are eligible for the first cancer screening (skin) at the age of 35 years while the first screenings (cervix) for women are much earlier at 20 years of age. However, only one male participant was aged below the screening age requirement. The location bias is also worth noting because many interviewees lived in or near Heidelberg, which is renowned for its world-class medical facilities.

Furthermore, we used slightly different primary questions in the protocol for interviewing current or former cancer patients. Unfortunately, a clear distinction could not be established whether the barriers pointed out by the patients/survivors’ group were related to screenings offers or treatment services. However, we have supplemented such findings by sample quotes to specify these differences. We employed a broad, wide-ranging approach to investigate all general factors of underutilization of screenings and treatments. Since the tests and techniques vary, specific barriers for each screening or treatment intervention could not be identified, which could be explored in the future. Moreover, further research employing either broader samples or purposive selection for underrepresented groups can be useful for overcoming the sample bias. Studies based on flexible data collection methods such as focus groups and ethnographic participant observation may also provide valuable insights into personal experiences of individuals and communities.

## Conclusions

This study explored the underlying reasons of low participation rates in early detection and cancer-related healthcare services in Germany. Based on personal interviews with current or former cancer patients and non-patients, we reported the barriers perceived and experienced by service users that might influence their decisions and behaviors about attending cancer-related health services. Most barriers could be assigned into three macro-level categories of (a) systemic inefficiencies within the German healthcare system; (b) personal beliefs, behaviors or attitudes; and (c) sociocultural norms and communication problems. Related to inefficiencies within the healthcare system, the most pertinent barriers were rooted in lack of information and awareness among the participants about the existing services. Further barriers stemmed from personal attitudes such as fear of cancer or the screening/treatment intervention itself and from sociocultural norms of gendered behavior, especially for men. To facilitate public participation in cancer-related healthcare services, a proactive approach by healthcare personnel and health insurance companies for information exchange with the general population is recommended. Service providers should identify the social risk factors and address the needs of populations eligible for screenings. Healthcare professionals are encouraged to introduce service users to cancer-related information at an earlier age and proactively inform them about available services. Our findings may be useful for policymakers and researchers to help design new or modify existing age- and gender-specific interventions and encourage public participation in cancer-related services. Further research to explore how governance and regulations create systemic inefficiencies that in turn impact health-seeking behaviors is warranted.

## Supplementary Information

Supplementary Material 1.

## Data Availability

The complete dataset used and/or analyzed for this current study are available from the corresponding author on reasonable request.

## References

[1] Bray F, Ferlay J, Soerjomataram I, Siegel RL, Torre LA, Jemal A. Global cancer statistics 2018: GLOBOCAN estimates of incidence and mortality worldwide for 36 cancers in 185 countries. Cancer J Clin. 2018;68(6):394–424. 10.3322/caac.21492 30207593

[2] Global Burden of Disease Cancer Collaboration. Global, regional, and National Cancer incidence, mortality, years of life lost, years lived with disability, and Disability-Adjusted life-Years for 29 Cancer groups, 1990 to 2017: A systematic analysis for the global burden of disease study. JAMA Oncol. 2019;5(12):1749–68. 31560378 10.1001/jamaoncol.2019.2996 PMC6777271

[3] Schiffman JD, Fisher PG, Gibbs P. Early detection of cancer: past, present, and future. Am Soc Clin Oncol Educ Book. 2015:57–65. 10.14694/EdBook_AM.2015.35.57. 10.14694/EdBook_AM.2015.35.57 25993143

[4] Institute of Medicine (US) & National Research Council (US). Fulfilling the Potential of Cancer Prevention and Early Detection. In: Curry SJ, Byers T, Hewitt M, editors. Washington, DC: The National Academies Press; 2003. p. 564. 10.17226/10263. 25057588

[5] Organisation for Economic Cooperation and Development & European Commission. EU country Cancer profile: Germany 2025. EU country Cancer profiles. Paris: Organisation for Economic Cooperation and Development; 2025. p. 3.

[6] German Federal Ministry of Health. Early detection of cancer: what screening is available? [https://gesund.bund.de/en/early-detection-of-cancer#examinations]

[7] Gemeinsamer Bundesausschuss. Richtlinie des Gemeinsamen Bundesausschusses für organisierte Krebsfrüherkennungsprogramme. In: Gemeinsamer Bundesausschuss, editor. 2022. Available at: https://www.g-ba.de/downloads/62-492-3039/oKFE-RL-2022-11-17-iK-2023-01-26.pdf.

[8] Muschol J, Strauss C, Gissel C. COVID-19 related decline in cancer screenings most pronounced for elderly patients and women in germany: a claims data analysis. J Cancer Res Clin Oncol. 2023;149(8):5345–67. 36436091 10.1007/s00432-022-04433-z PMC9702775

[9] Starker A, Buttmann-Schweiger N, Krause L, Barnes B, Kraywinkel K, Holmberg C. Krebsfrüherkennungsuntersuchungen in deutschland: Angebot und inanspruchnahme. Bundesgesundheitsblatt - Gesundheitsforschung - Gesundheitsschutz. 2018;61(12):1491–9. 30406892 10.1007/s00103-018-2842-8

[10] Augustin M, Stadler R, Reusch M, Schäfer I, Kornek T, Luger T. Skin cancer screening in Germany - perception by the public. J Der Deutschen Dermatologischen Gesellschaft = J German Soc Dermatology: JDDG. 2012;10(1):42–9. 10.1111/j.1610-0387.2011.07761.x 21923730

[11] Eissing L, Schäfer I, Strömer K, Kaufmann R, Enk A, Reusch M, Augustin M. Perception of statutory skin cancer screening in the general population: current findings on participation, knowledge and evaluation. Der Hautarzt; Z fur Dermatologie Venerologie Und Verwandte Gebiete. 2017;68(5):371–6. 10.1007/s00105-017-3943-2 28246676

[12] Görig T, Schneider S, Schilling L, Diehl K. Barriers to using a nationwide skin Cancer screening program: findings from Germany. Oncol Res Treat. 2018;41(12):774–9. 30458453 10.1159/000492440

[13] Starker A, Kraywinkel K, Kuhnert R. Früherkennung von brustkrebs: inanspruchnahme der mammografie in Deutschland. J Health Monit. 2017;2(4):74–80.

[14] Starker A, Buttmann-Schweiger N, Kraywinkel K, Kuhnert R. Inanspruchnahme der darmspiegelung in Deutschland. J Health Monit. 2017;2(4):81–7. 10.17886/RKI-GBE-2017-126 PMC10165909 37168126

[15] US Preventive Services Task Force. Screening for lung cancer: US preventive services task force recommendation statement. JAMA. 2021;325(10):962–70. 33687470 10.1001/jama.2021.1117

[16] US Preventive Services Task Force. Screening for colorectal cancer: US preventive services task force recommendation statement. JAMA. 2021;325(19):1965–77. 34003218 10.1001/jama.2021.6238

[17] US Preventive Services Task Force. Screening for cervical cancer: US preventive services task force recommendation statement. JAMA. 2018;320(7):674–86. 30140884 10.1001/jama.2018.10897

[18] US Preventive Services Task Force. Screening for breast cancer: US preventive services task force recommendation statement. JAMA. 2024;331(22):1918–30. 38687503 10.1001/jama.2024.5534

[19] National Institute for Public Health and the Environment. Participation rates in cancer population screening programmes continue to decline. National Institute for Public Health and the Environment; 2023. Available at: https://www.rivm.nl/en/news/participation-rates-in-cancer-population-screening-programmes-continue-to-decline.

[20] Dryden R, Williams B, McCowan C, Themessl-Huber M. What do we know about who does and does not attend general health checks? Findings from a narrative scoping review. BMC Public Health. 2012;12:723. 22938046 10.1186/1471-2458-12-723 PMC3491052

[21] Maliski SL, Connor SE, Oduro C, Litwin MS. Access to health care and quality of life for underserved men with prostate cancer. Semin Oncol Nurs. 2011;27(4):267–77. 22018406 10.1016/j.soncn.2011.07.005

[22] Edwards DJ, Sakellariou D, Anstey S. Barriers to, and facilitators of, access to cancer services and experiences of cancer care for adults with a physical disability: A mixed methods systematic review. Disabil Health J. 2020;13(1):100844. 31668781 10.1016/j.dhjo.2019.100844

[23] Fernandez ME, Wippold R, Torres-Vigil I, Byrd T, Freeberg D, Bains Y, Guajardo J, Coughlin SS, Vernon SW. Colorectal Cancer screening among Latinos from U.S. Cities along the Texas-Mexico border. Cancer Causes Control. 2008;19(2):195–206. 18038186 10.1007/s10552-007-9085-6

[24] Mojica CM, Parra-Medina D. Interventions promoting colorectal Cancer screening among Latino men: A systematic review. Prev Chronic Dis. 2018;15:170218. 10.5888/pcd15.170218 PMC5858157 29522700

[25] Quaglia A, Vercelli M, Lillini R, Mugno E, Coebergh JW, Quinn M, Martinez-Garcia C, Capocaccia R, Micheli A, Group EW. Socio-economic factors and health care system characteristics related to cancer survival in the elderly: A population-based analysis in 16 European countries (ELDCARE project). Crit Rev Oncol/Hematol. 2005;54(2):117–28. 15843094 10.1016/j.critrevonc.2004.12.001

[26] Batouli A, Jahanshahi P, Gross CP, Makarov DV, Yu JB. The global cancer divide: relationships between National healthcare resources and cancer outcomes in high-income vs. middle- and low-income countries. J Epidemiol Global Health. 2014;4(2):115–24. 10.1016/j.jegh.2013.10.004 PMC7366371 24857179

[27] Rosenstock IM. Historical origins of the health belief model. Health Educ Monogr. 1974;2(4):328–35. 10.1177/109019817800600406 299611

[28] Leone JE, Rovito MJ. Normative content and health inequity enculturation: A logic model of men’s health advocacy. Am J Men’s Health. 2013;7(3):243–54. 23283809 10.1177/1557988312469659

[29] Baer HA, Singer M, Johnsen JH. Toward a critical medical anthropology. Soc Sci Med. 1986;23(2):95–8. 3749976

[30] Lim WM. What Is Qualitative Research? An Overview and Guidelines. Australas Mark J. 2024;33(2):199–229. 10.1177/14413582241264619.

[31] Purna Singh A, Vadakedath S, Kandi V. Clinical research: A review of study designs, hypotheses, errors, sampling types, ethics, and informed consent. Cureus. 2023;15(1):e33374. 36751199 10.7759/cureus.33374 PMC9898800

[32] World Medical Association. World Medical Association Declaration of Helsinki: ethical principles for medical research involving human participants. JAMA. 2025;333(1):71–4. 10.1001/jama.2024.21972. 39425955 10.1001/jama.2024.21972

[33] Tong A, Sainsbury P, Craig J. Consolidated criteria for reporting qualitative research (COREQ): a 32-item checklist for interviews and focus groups. Int J Qual Health Care. 2007;19(6):349–57. 17872937 10.1093/intqhc/mzm042

[34] Scott SE, Walter FM, Webster A, Sutton S, Emery J. The model of pathways to treatment: conceptualization and integration with existing theory. Br J Health Psychol. 2013;18(1):45–65. 22536840 10.1111/j.2044-8287.2012.02077.x

[35] Niederberger M, Köberich S, Network motD. Coming to consensus: the Delphi technique. Eur J Cardiovasc Nurs. 2021;20(7):692–5. 34245253 10.1093/eurjcn/zvab059

[36] Kuckartz U, Rädiker S. Die Interviews codieren („Basiscodierung). In: Fokussierte Interview analyse mit MAXQDA: Schritt für Schritt. Kuckartz U, Rädiker S, editors. Wiesbaden: Springer Fachmedien Wiesbaden; 2020. p. 43–54. https://www.springerprofessional.de/die-interviews-codieren-basiscodierung/27601988.

[37] Daniati N, Widjaja G, Olalla M, Chaudhary P, Shalaby M, Chupradit S, Mustafa Y. The health belief model’s application in the development of health behaviors. Health Educ Health Promotion. 2022;9:521–7.

[38] Braveman P, Gottlieb L. The social determinants of health: it’s time to consider the causes of the causes. Public Health Rep. 2014;129(Suppl 2):19–31. 24385661 10.1177/00333549141291S206 PMC3863696

[39] Bozhar H, McKee M, Spadea T, Veerus P, Heinävaara S, Anttila A, Senore C, Zielonke N, de Kok IMCM, van Ravesteyn NT, et al. Socio-economic inequality of utilization of cancer testing in europe: A cross-sectional study. Prev Med Rep. 2022;26:101733. 35198362 10.1016/j.pmedr.2022.101733 PMC8850331

[40] Wu Z, Liu Y, Li X, Song B, Ni C, Lin F. Factors associated with breast cancer screening participation among women in Mainland china: a systematic review. BMJ Open. 2019;9(8):e028705. 31455705 10.1136/bmjopen-2018-028705 PMC6720337

[41] Rehman S, Lim M, Sidhu R, Ramis P, Rohren E. Barriers to lung cancer screening. Cancer Epidemiol. 2025;94:102722. 39647346 10.1016/j.canep.2024.102722

[42] Adunlin G, Cyrus JW, Asare M, Sabik LM. Barriers and facilitators to breast and cervical Cancer screening among immigrants in the united States. J Immigr Minor Health. 2019;21(3):606–58. 30117005 10.1007/s10903-018-0794-6

[43] Al Daragemeh AI, Saleh AM, Abdel-Aziz HR, V A, AlOmari AK, AlOmari AA. From Insights to Impact: Understanding Cancer Screening Choices through Mixed-Methods. Asian Pac J Cancer Prev. 2024;25(8):2655–60. 10.31557/APJCP.2024.25.8.2655. 10.31557/APJCP.2024.25.8.2655 PMC11495428 39205562

[44] Teo CH, Ng CJ, Booth A, White A. Barriers and facilitators to health screening in men: A systematic review. Soc Sci Med. 2016;165:168–76. 27511617 10.1016/j.socscimed.2016.07.023

[45] Starker A, Saß AC. Inanspruchnahme von Krebsfrüherkennungsuntersuchungen. Bundesgesundheitsblatt - Gesundheitsforschung - Gesundheitsschutz. 2013;56(5):858–67. 23703507 10.1007/s00103-012-1655-4

[46] Fox SA, Heritage J, Stockdale SE, Asch SM, Duan N, Reise SP. Cancer screening adherence: does physician–patient communication matter? Patient Educ Couns. 2009;75(2):178–84. 19250793 10.1016/j.pec.2008.09.010

[47] Peterson EB, Ostroff JS, DuHamel KN, D’Agostino TA, Hernandez M, Canzona MR, Bylund CL. Impact of provider-patient communication on cancer screening adherence: A systematic review. Prev Med. 2016;93:96–105. 27687535 10.1016/j.ypmed.2016.09.034 PMC5518612

[48] Fochler S, Weitmann K, Domin M, Hoffmann W. Reasons for Non-Attendance in the German National mammography screening program: which barriers can be overcome using telephone Counseling?-A randomized controlled trial. Healthcare (Basel). 2022;11(1). 10.3390/healthcare11010017. 10.3390/healthcare11010017 PMC9818884 36611477

[49] Allgood PC, Maroni R, Hudson S, Offman J, Turnbull AE, Peacock L, Steel J, Kirby G, Ingram CE, Somers J, et al. Effect of second timed appointments for non-attenders of breast cancer screening in england: a randomised controlled trial. Lancet Oncol. 2017;18(7):972–80. 28522311 10.1016/S1470-2045(17)30340-6 PMC5489696

[50] Zhang X, Elsaid MI, DeGraffinreid C, Champion VL, Paskett ED. Impact of the COVID-19 pandemic on Cancer screening delays. J Clin Oncol. 2023;41(17):3194–202. 36735899 10.1200/JCO.22.01704 PMC10256430

[51] Duffy SW, Seedat F, Kearins O, Press M, Walton J, Myles J, Vulkan D, Sharma N, Mackie A. The projected impact of the COVID-19 lockdown on breast cancer deaths in England due to the cessation of population screening: a National Estimation. Br J Cancer. 2022;126(9):1355–61. 35110696 10.1038/s41416-022-01714-9 PMC8808468

[52] Corley DA, Sedki M, Ritzwoller DP, Greenlee RT, Neslund-Dudas C, Rendle KA, Honda SA, Schottinger JE, Udaltsova N, Vachani A, et al. Cancer screening during the coronavirus Disease-2019 pandemic: A perspective from the National Cancer institute’s PROSPR consortium. Gastroenterology. 2021;160(4):999–1002. 33096099 10.1053/j.gastro.2020.10.030 PMC7575503

[53] Kannan A, Kirkman M, Ruseckaite R, Evans SM. Prostate care and prostate cancer from the perspectives of undiagnosed men: a systematic review of qualitative research. BMJ Open. 2019;9(1):e022842. 30782686 10.1136/bmjopen-2018-022842 PMC6352751

[54] Medina-Perucha L, Yousaf O, Hunter MS, Grunfeld EA. Barriers to medical help-seeking among older men with prostate cancer. J Psychosoc Oncol. 2017;35(5):531–43. 28368770 10.1080/07347332.2017.1312661

[55] Knight JR, Williamson LH, Armstrong DK, Westbrook EA. Understanding lung Cancer resources and barriers among worksites with mostly male employees in eight rural Kentucky counties: A focus group discussion. Am J Men’s Health. 2019;13(6):1557988319882585–1557988319882585. 31703543 10.1177/1557988319882585 PMC6843751

[56] Lee DJ, Consedine NS, Spencer BA. Barriers and facilitators to digital rectal examination screening among African-American and African-Caribbean men. Urology. 2011;77(4):891–8. 21477716 10.1016/j.urology.2010.11.056 PMC3092791

[57] Zeissig SR, Singer S, Koch L, Blettner M, Arndt V. [Utilisation of Psycho-oncological services in hospitals and outpatient counselling centres by survivors of breast, Colon and prostate cancer]. Psychother Psychosom Med Psychol. 2015;65(5):177–82. 25485601 10.1055/s-0034-1395627

[58] Mitchell AJ. Screening for cancer-related distress: when is implementation successful and when is it unsuccessful? Acta Oncol. 2013;52(2):216–24. 23320770 10.3109/0284186X.2012.745949

[59] Leppin N, Nagelschmidt K, Koch M, Riera-Knorrenschild J, Seifart C, Rief W, Barke A, von Blanckenburg P. Cancer patient utilisation of psychological care in germany: the role of attitudes towards seeking help. Eur J Cancer Care. 2019;28(6):e13165. 10.1111/ecc.13165 31571288

[60] Carolan CM, Smith A, Davies GR, Forbat L. Seeking, accepting and declining help for emotional distress in cancer: A systematic review and thematic synthesis of qualitative evidence. Eur J Cancer Care (Engl). 2018;27(2):e12720. 28597493 10.1111/ecc.12720

[61] Tondorf T, Grossert A, Rothschild SI, Koller MT, Rochlitz C, Kiss A, Schaefert R, Meinlschmidt G, Hunziker S, Zwahlen D. Focusing on cancer patients’ intentions to use Psychooncological support: A longitudinal, mixed-methods study. Psychooncology. 2018;27(6):1656–63. 29656415 10.1002/pon.4735 PMC6001470

[62] Hajek A, Bock J-O, König H-H. The use of routine health check-ups and psychological factors—a neglected link. Evidence from a population-based study. J Public Health. 2018;26(2):137–44.

[63] Brunner-Ziegler S, Rieder A, Stein KV, Koppensteiner R, Hoffmann K, Dorner TE. Predictors of participation in preventive health examinations in Austria. BMC Public Health. 2013;13(1):1138. 24308610 10.1186/1471-2458-13-1138 PMC3866300

[64] Schülein S, Taylor KJ, Schriefer D, Blettner M, Klug SJ. Participation in preventive health check-ups among 19,351 women in Germany. Prev Med Rep. 2017;6:23–6. 28210539 10.1016/j.pmedr.2017.01.011 PMC5310169

[65] Dorak MT, Karpuzoglu E. Gender differences in cancer susceptibility: an inadequately addressed issue. Front Genet. 2012;3:268–268. 23226157 10.3389/fgene.2012.00268 PMC3508426

[66] Thompson L, Reeder T, Abel G. I can’t get my husband to go and have a colonoscopy’: gender and screening for colorectal cancer. Health. 2011;16(3):235–49. 21602246 10.1177/1363459311403948

[67] Singer S, Wünsch A, Ihrig A, Bruns G, Holz F, Jakob J, Besseler M, Engesser D, Blettner M, König J, et al. Men’s access to outpatient psychosocial Cancer counseling. Dtsch Arztebl Int. 2024;121(4):121–7. 38231700 10.3238/arztebl.m2024.0005 PMC11019762

[68] Lee DJ, Consedine NS, Gonzalez JR, Spencer BA. Association of healthcare barriers with Prostate-specific antigen screening among African-American and Afro-Caribbean men. Urology. 2012;80(3):556–63. 22789295 10.1016/j.urology.2012.02.085

[69] Okuyama T, Endo C, Seto T, Kato M, Seki N, Akechi T, Furukawa TA, Eguchi K, Hosaka T. Cancer patients’ reluctance to disclose their emotional distress to their physicians: a study of Japanese patients with lung cancer. Psycho-oncology. 2008;17(5):460–5. 17828705 10.1002/pon.1255

[70] Henselmans I, Jacobs M, van Berge Henegouwen MI, de Haes HC, Sprangers MA, Smets EM. Postoperative information needs and communication barriers of esophageal cancer patients. Patient Educ Couns. 2012;88(1):138–46. 22244819 10.1016/j.pec.2011.12.004

[71] Brandes K, Linn AJ, Smit EG, van Weert JCM. Patients’ reports of barriers to expressing concerns during cancer consultations. Patient Educ Couns. 2015;98(3):317–22. 25499005 10.1016/j.pec.2014.11.021

[72] Singh Ospina N, Phillips KA, Rodriguez-Gutierrez R, Castaneda-Guarderas A, Gionfriddo MR, Branda ME, Montori VM. Eliciting the patient’s Agenda- secondary analysis of recorded clinical encounters. J Gen Intern Med. 2019;34(1):36–40. 29968051 10.1007/s11606-018-4540-5 PMC6318197

