# Supplementary material for: ‘I don’t know when it’s my turn’: reasons for low uptake of cancer-related healthcare services in Germany
Source: BMC Public Health. 2025 Jul 19;25:2505. doi: 10.1186/s12889-025-23656-6 (PMC12275257; doi:10.1186/s12889-025-23656-6)
Supplement: Supplementary file 1 — Supplementary Material 1. [file 12889_2025_23656_MOESM1_ESM.docx]

**COREQ-32**

**Consolidated criteria for reporting qualitative studies (COREQ): 32-item checklist**

|  | **Item** | **Guide questions/description** | **Reported (Page number)** |
| --- | --- | --- | --- |
| **Domain 1: Research team and reflexivity** | | | |
| **Personal Characteristics** | | | |
| 1. | Interviewer/facilitator | Which author/s conducted the interview or focus group? | MK conducted all semi-structured interviews. (Page 4) |
| 2. | Credentials | What were the researcher's credentials? *E.g. PhD, MD* | M.A. in sociocultural anthropology, and several years of experience in social work. (Page 4) |
| 3. | Occupation | What was their occupation at the time of the study? | MK worked as a research associate in the study team and was also employed as a social worker working with the city council. (Page 4) |
| 4. | Gender | Was the researcher male or female? | Female. (Page 4) |
| 5. | Experience and training | What experience or training did the researcher have? | As a social worker, MK routinely conducts interviews and focus groups with migrants in Germany. She completed her master’s in anthropology with training on qualitative methods and ethnography. (Page 4) |
| **Relationship with participants** | | | |
| 6. | Relationship established | Was a relationship established prior to study commencement? | The research team did not have contact with participants prior to emailing them to invite for the interviews. (Page 4) |
| 7. | Participant knowledge of the interviewer | What did the participants know about the researcher? e*.g. personal goals, reasons for doing the research* | The study objectives were described in the information leaflet, which all participants had read and agreed to in the first phase of the study. They completed a questionnaire and agreed to be contacted for an interview at a later stage. In the subsequent invitation email for interviews, the participants were informed about the researcher who will be conducting their interviews. (Page 4) |
| 8. | Interviewer characteristics | What characteristics were reported about the interviewer/facilitator? e.g. *Bias, assumptions, reasons and interests in the research topic* | The research team aimed to explore the barriers to attending cancer related services among the general population, assuming certain reasons behind the underutilization of cancer-related health services. (Page 4) |
| **Domain 2: Study design** | | | |
| **Theoretical framework** | | | |
| 9. | Methodological orientation and theory | What methodological orientation was stated to underpin the study? *e.g. grounded theory, discourse analysis, ethnography, phenomenology, content analysis* | The research team conducted content analysis and analyzed the data using the hybrid inductive-deductive methodology described by Udo Kuckartz. (Page 5) |
| **Participant selection** | | | |
| 10. | Sampling | How were participants selected? *e.g. purposive, convenience, consecutive, snowball* | Interviewees were recruited using voluntary response sampling of the participants (N=57), who had agreed to be interviewed in the quantitative stage of the study. (Page 4) |
| 11. | Method of approach | How were participants approached? e*.g. face-to-face, telephone, mail, email* | Invitation emails were sent to participants, who had agreed to be interviewed in the first stage of the study, for an online interview. The interviews were conducted via online video conferencing and, in some cases, via telephone. (Page 4) |
| 12. | Sample size | How many participants were in the study? | Overall 23 interviews were conducted with 14 male and 9 female participants. (Page 5) |
| 13. | Non-participation | How many people refused to participate or dropped out? Reasons? | Overall 57 participants consented to be interviewed. 20 individuals did not respond to invitation emails. 2 email addresses were unreachable. 11 participants refused to be interviewed. 1 participant did not join the interview on the agreed date. (Page 4) |
| **Setting** | | | |
| 14. | Setting of data collection | Where was the data collected? e*.g. home, clinic, workplace* | Interviews were conducted and recorded on workplace computers. (Page 4) |
| 15. | Presence of non-participants | Was anyone else present besides the participants and researchers? | No. (Page 4) |
| 16. | Description of sample | What are the important characteristics of the sample? *e.g. demographic data, date* | The interviewees included 14 men and 9 women with mean age of 58 years. The youngest participant was 23 years old and the oldest was 82 years old. There were 13 cancer patients/survivors and 10 non-patients.  The interviews were conducted between January 2022 and February 2023. (Page 5) |
| **Data collection** | | | |
| 17. | Interview guide | Were questions, prompts, guides provided by the authors? Was it pilot tested? | The participants did not receive the questions or prompts before the interview. The interview protocol was piloted within the research group and finalized after two Delphi rounds. (Page 4) |
| 18. | Repeat interviews | Were repeat interviews carried out? If yes, how many? | There were no repeat interviews with the same participants. (Page 4) |
| 19. | Audio/visual recording | Did the research use audio or visual recording to collect the data? | All interviews were audio recorded with the permission of participants. (Page 4) |
| 20. | Field notes | Were field notes made during and/or after the interview or focus group? | The researcher made personal field notes during the interviews that were not analyzed during data analysis. (Page 4) |
| 21. | Duration | What was the duration of the interviews or focus group? | The interviews lasted between 30 and 45 minutes. (Page 4) |
| 22. | Data saturation | Was data saturation discussed? | Interviews were conducted using voluntary response sampling and the recruitment methodology did not allow data saturation. Saturation points were discussed during data analysis and the authors agreed on the derived codes from inductive coding technique. (Page 4 & 5) |
| 23. | Transcripts returned | Were transcripts returned to participants for comment and/or correction? | Transcripts were not returned to participants for comments and/or correction. (Page 4) |
| **Domain 3: Analysis and findings** | | | |
| **Data analysis** | | | |
| 24. | Number of data coders | How many data coders coded the data? | Two researchers (LW & NT) coded two interviews to create the preliminary deductive coding tree from the interview protocol. All interviews were inductively coded by both researchers independently with regular feedback from other authors. After completing inductive coding tree, AZ, LW & NT reached 100% consensus on coding. (Page 5) |
| 25. | Description of the coding tree | Did authors provide a description of the coding tree? | The coding tree is available in supplementary material. (Page 5) |
| 26. | Derivation of themes | Were themes identified in advance or derived from the data? | Themes were derived from the data using a hybrid deductive-inductive model. (Page 5) |
| 27. | Software | What software, if applicable, was used to manage the data? | MAXQDA 2022. (Page 5) |
| 28. | Participant checking | Did participants provide feedback on the findings? | No. (Page 4) |
| **Reporting** | | | |
| 29. | Quotations presented | Were participant quotations presented to illustrate the themes / findings? Was each quotation identified? e*.g. participant number* | Key findings of this study are supported with selected quotes within the manuscript text with participant codes. Additional supporting quotes are provided in supplementary material. (Page 5) |
| 30. | Data and findings consistent | Was there consistency between the data presented and the findings? | All findings were derived from the data and all themes are supported by illustrative quotes. (Page 5-9) |
| 31. | Clarity of major themes | Were major themes clearly presented in the findings? | Major themes derived from the data are clearly labelled under a paragraph headline. (Page 5-9) |
| 32. | Clarity of minor themes | Is there a description of diverse cases or discussion of minor themes? | The authors chose to include only the categories most pertinent to the research question and with highest prevalence in content analysis for this publication. All themes are presented in the supplementary material. (Page 5) |
